# Effects of a Multimodal Program on Frailty Syndrome and Psychological Alterations in Breast Cancer Women Treated with Aromatase Inhibitors

**DOI:** 10.3390/clinpract15030041

**Published:** 2025-02-21

**Authors:** Pedro Céspedes, Francisco M. Martínez-Arnau, María Dolores Torregrosa, Omar Cauli, Cristina Buigues

**Affiliations:** 1 Department of Nursing, University of Valencia, 46010 Valencia, Spain; pedro.cespedes@uv.es (P.C.); cristina.buigues@uv.es (C.B.); 2 Department of Physiotherapy, University of Valencia, 46010 Valencia, Spain; francisco.m.martinez@uv.es; 3 Frailty and Cognitive Impairment Research Group (FROG), University of Valencia, 46010 Valencia, Spain; 4 Medical Oncology Department, Doctor Peset University Hospital, 46017 Valencia, Spain; torregrosa_dol@gva.es

**Keywords:** multimodal program, physical exercise, health education, breast cancer, postmenopause, aromatase inhibitors, hormone therapy, frailty syndrome, mental health, depressive symptoms, anxious symptoms, insomnia

## Abstract

**Background/Objectives:** Treatment with aromatase inhibitors can worsen frailty syndrome and psychological symptoms in women diagnosed with breast cancer (BC) receiving these drugs to prevent cancer recurrence. We analyze whether postmenopausal women with localized BC receiving aromatase inhibitors (AIs) treatment can achieve improvements in their mental health and their level of frailty through a multimodal program that includes supervised physical exercise and health education workshops. **Methods:** A total of 52 postmenopausal women with a prior diagnosis of BC and receiving hormonal treatment with AIs were included in the multimodal physical exercise and health education program and evaluated before and after it. The assessment included the following five frailty syndrome (FS) criteria: involuntary weight loss, weakness, low physical activity, slow gait speed, and low muscle strength. Mental health was assessed using the Goldberg scale, with its subscales for anxiety and depressive symptoms. The Athens scale was used to assess subjective sleep quality. **Results:** There was a significant difference in the number of robust, pre-frail and frail women after the program compared to the baseline. Six women did not fulfill any criteria for (robust) FS before the program (11.5%), and thirty-three women (63.5%) after the program did not fulfill any criteria for FS. A total of 33 (63.5%) women met one or two FS criteria (pre-frail) before the program, and 18 (34.6%) met one or two FS criteria after the program; thirteen (25%) women met three or more FS criteria (frail) before the program and one (1.9%) after it (*p* < 0.001). A statistically significant improvement on the Goldberg scale was observed (on both the subscales for anxiety and depressive symptoms) (*p* < 0.001). A statistically significant improvement was also noted on the Athens insomnia scale (*p* < 0.001). A multivariate regression model analysis identified marital status (being married) (*p* = 0.047, beta coefficient= −0.249, 95% CI −1.4844–−0.14) and the percentage of attendance at training sessions (*p* = 0.041, beta coefficient = −0.290, 95% CI 0.104–0.002) as associated variables, with a lower score on the Goldberg depression subscale. **Conclusions:** Mental health and frailty, common in postmenopausal women diagnosed with BC on hormonal treatment with AI, can be improved with multimodal programs of supervised physical exercise and health education.

## 1. Introduction

Breast cancer (BC) is the most prevalent cancer among women worldwide [1]. Improved diagnostic along with therapeutic interventions in recent decades and, therefore, improved prognosis have led to an increase in the prevalence of BC in older patients [2]. These older women account for one-third of BC cases [3], while most estrogen-receptor-positive (ER+) breast tumors are found in postmenopausal women (~80%) [4]. Aromatase inhibitors (AIs) are the first-line adjuvant treatment to reduce cancer recurrence in postmenopausal patients with estrogen-receptor-positive localized breast cancer [5]. These drugs are prescribed for at least 5 years after radical cancer treatment and sometimes for up to 10 years [6]. Recent clinical trials have shown AIs to be more effective than tamoxifen in reducing cancer recurrence, and they are considered a very useful and extensively used drug [7].

Although AIs play a key role in increasing survival and reducing relapses [8], they are not free of unwanted adverse effects, in addition to those of other oncological treatments and the comorbidities that tend to be especially predominant in these patients [8,9,10]. Recent studies have shown that treatment with AIs is associated with increased levels of frailty syndrome in postmenopausal women diagnosed with BC [11]. Frailty syndrome (FS) is defined as a potentially reversible health state characterized by impairment of multiple physiological systems [12,13]. As a consequence of the severe reduction in estrogen levels caused by this treatment with AIs, postmenopausal women have a high risk of deterioration in these levels of frailty [11].

Furthermore, AIs can also cause or worsen depressive and anxiety symptoms in these patients [10,14,15], which may contribute to poor adherence to drug treatment. These unwanted adverse effects cause significant discomfort to patients and may lead to poorer adherence to this treatment [16,17,18,19]. AIs can reduce subjective sleep quality as a consequence of the reduction in estrogens’ positive sleep regulation [10,20,21].

Frailty and psychological alterations partially overlap and may influence each other. Frailty syndrome is associated with depressive and anxiety symptoms and insomnia in older patients diagnosed with breast cancer who are receiving hormonal therapy, which is increased by the impact of AI treatment [10,22,23,24,25,26]. Previous studies have explored the relationships between cognitive and emotional state and physical frailty, demonstrating a bidirectional association between the two domains. Psychological symptoms are thought to worsen the degree of physical frailty, and physical frailty itself poses a risk of a worsened cognitive state and depression [13,27].

The quality of life of women diagnosed with BC on AIs, influenced by the physical and psychological adverse effects that this type of hormonal treatment can induce, can be improved by regular physical exercise, especially when supervised [28]. Similarly, the accumulation of functional decline in older cancer patients and the high levels of frailty that often accompany them can be controlled and reduced by physical exercise [24]. In this sense, different exercise interventions have managed, in addition to improving their levels of physical and mental health, to improve muscle strength and reduce fatigue perceived by patients diagnosed with BC [24,29], mitigating the negative impact of these hormonal treatments with AIs [28].

These types of interventions may help improve cancer care to attenuate frailty-related outcomes while extending their benefits to the overall health of this patient group [24,29]. The evidence found supports the need for further research on this topic and the evaluation of rigorously structured exercise interventions (especially in the multimodal format with health education sessions) [24]. Adjuvant treatment with AIs is well demonstrated to prevent cancer relapses, but because the treatment lasts 5 to 10 years, adherence may be suboptimal due to the side effects these drugs generate. Health education is crucial not only for self-management but also for the prevention, recognition, and treatment of adverse effects, which are of the utmost importance in promoting adherence [15]. No trials have assessed the effect of a multimodal intervention based on combined physical exercise and health education workshops on frailty syndrome in older breast cancer patients receiving AI-adjuvant treatment. Besides physical exercise, it may be necessary to implement and adapt the designs of educational interventions to the age of patients, preparing them for the adverse effects that the diagnosis and treatment of breast cancer have on social, physical, and functional levels and to achieve a reduction in anxiety levels in this group of patients, increasing their knowledge and improving their self-management [30,31,32]. Different types of educational intervention appear to afford positive results on anxiety, depression, psychological distress, patient knowledge, and pain in older patients with cancer [30].

Even if it is well-known that a decrease in estrogen levels can induce or worsen frailty [33,34,35] or psychological alterations in older women [33,34,35,36,37] and that treatment with AIs in breast cancer can lead to or worsen frailty syndrome [11] and psychological alterations such as depressive symptoms and insomnia [10,38,39], to date, there is no trial on the reversal of these alterations in women with breast cancer under AIS treatment within a multimodal intervention program.

We tested whether a multimodal intervention based on supervised physical exercise and a health education program can improve frailty, anxiety, depressive symptoms, and sleep quality in women with localized breast cancer treated with aromatase inhibitors used in an adjuvant setting in order to prevent cancer relapse. As secondary outcomes, we plan to determine women’s adherence to the program, the relationship between the time since diagnosis and the start of AI treatment with the primary outcomes, and the relationship between clinical and sociodemographic variables and these primary outcomes.

## 2. Materials and Methods

### 2.1. Study Design

A longitudinal study was conducted in postmenopausal women with estrogen-positive localized breast cancers under treatment with AIs, who were treated and followed up in the Medical Oncology Service of Doctor Peset University Hospital (Valencia, Spain). For the study, a complete pre-assessment of frailty levels, anxiety and depressive symptoms, and sleep quality was carried out in this hospital service. This was followed by a multimodal intervention of physical exercise and health education. Finally, a post-program assessment was carried out. The study received approval from the clinical research ethics committee of the Doctor Peset University Hospital (CEIm code: 102.22 approval date 4 November 2022).

### 2.2. Participants and Setting

Oncologists in the Oncology Service of the Doctor Peset University Hospital in Valencia identified and recruited women consecutively as they visited the hospital for medical evaluations between September 2023 and April 2024. The participants had to meet the following criteria to be eligible for and take part in the intervention: be 60 years of age or older, diagnosed with localized hormone-dependent breast cancer, have undergone surgery, and receive adjuvant treatment with one of the AIs. In addition, to participate in the program, they had to have one of the criteria of FS and/or exceed the cut-off point for a clinically relevant diagnosis according to the selected anxiety or depression scales.

The exclusion criteria were having a cognitive impairment or mental illness that limited the individual’s understanding of the health education workshops and having any physical/functional limitation that prevented travel and/or the performance of the physical exercise proposed in the program.

This study was undertaken with a convenience sample. To identify a minimum “a priori” sample size, we select the improvement of frailty syndrome as the main outcome of the intervention in older patients with breast cancer. For this calculation, we used previously published data reporting a 64% prevalence of frailty syndrome (pre-frailty and frailty pooled together) in older women with localized breast cancer under AIs [23]. Accepting an alpha risk of 0.05 and a power of 0.8 in a two-tailed test, 51 women were necessary to recognize a difference, with an initial prevalence of frailty syndrome of 64% and a final prevalence after the intervention of half of that at baseline (32%) as statistically significant.

### 2.3. Intervention and Follow-Up: Multimodal Program

The program was carried out in three phases: the pre-intervention assessment, the execution of the intervention, and the post-intervention assessment. The nurse explained the complementary information provided by the oncologists to all the women who met the inclusion criteria and formally invited them for an initial assessment. The characteristics, duration, type of exercise, and content of the educational sessions were explained at this point. After the informed consent was signed by the participants in the Oncology service of the Doctor Peset University Hospital, the nursing staff conducted a prior assessment interview in order to compile sociodemographic variables, compare the clinical information from the participant’s medical records, and assess their levels of mental health and subjective sleep quality using the selected scales and questionnaires. In addition, this assessment enabled the evaluation of the criteria of the FS that are self-administered. In order to adapt the program to the participants’ specific capacities, the physiotherapist, assisted by a graduate in physical education and a specialist in physical exercise, assessed their individual capacities by administering a series of physical tests in the place where they subsequently carried out the exercise sessions. Both the assessment interview by the nursing staff and the physical tests were repeated after the intervention had been carried out.

The multimodal program consisted of supervised sessions of physical exercise and health education. The multimodal physical exercise and health education intervention consisted of 12 weeks of combined physical training, with two 60 min sessions per week and six educational workshops on topics related to HT, BC, and healthy habits, each lasting 90 min.

The physical exercise was carried out in a senior center managed by Valencia City Council, alternating between outdoors and indoors depending on the weather conditions. This branch of the program was designed by a physiotherapist, coordinated by a nurse, and supervised and executed with the help of a graduate in education and a specialist. The participants were advised to adapt their physical abilities to the exercise in order to avoid injury and/or the aggravation of previous injuries. The exercise consisted of a multi-component community exercise program supervised by a trained physiotherapist [40,41]. The sessions consisted of three different sections. In each physical exercise session, the women were subjected for 5–10 min to a physical activity aimed at warming up muscles and promoting joint mobility. Then, the main part of the session (approximately 50 min in duration) consisted of three sets of physical exercise sessions at the lower and upper extremity level using elastic bands, followed by a 3 min break with active rest based on aerobic exercises (balance and/or cardiovascular). To end the session, physical cooling exercises (approximately 3–5 min) were performed with static stretching [42]. Throughout the program sessions, a progressive adaptation of the intensity of the physical exercise was carried out, taking as a reference the self-perceived effort that the patients perceived individually and giving a score from 0 to 10, with 0 being no effort and 10 being their maximum effort. Based on these results, the objective was to perform two weeks of acclimatization (exercise intensity 4–5 out of 10), three weeks at higher intensity (7 out of 10), and, finally, alternate weeks at maximum intensity (9–10 out of 10) and high intensity (7–8 out of 10).

The nurse taught four of the six health education sessions, specifically those on nutrition, mental health, pain, and insomnia. The oncologist taught a session on hormonal treatment and its adverse effects and self-care. The remaining session on physical exercise was taught by the physiotherapist. The objective of these sessions was to inform and train the participants on topics directly or indirectly related to their oncological diagnosis, treatments received (especially hormonal treatment), and healthy habits in order to empower them in their development of strategies to improve self-care. All the sessions included time for discussion and the exchange of experiences, opinions, and queries.

### 2.4. Data Collection

The pre-intervention and post-intervention evaluations included the following sections:

#### 2.4.1. Sociodemographic and Clinical Variables

From the pre-intervention assessments carried out in the Oncology Service of the Doctor Peset University Hospital, the following sociodemographic variables were obtained and analyzed: age, marital status, level of education, employment and cohabitation status, quantity and type of medication consumed not related to cancer treatment, and amount and type of physical exercise usual prior to the intervention. Marital status was dichotomized for bivariate and multivariate analyses. Disease and treatment characteristics were also obtained and analyzed. These were the time since diagnosis, whether or not they were treated with radiotherapy and chemotherapy, type of surgery, type of AI, and time of treatment with AI in months. Of these clinical variables, the type of surgery and staging were also dichotomized for bivariate and multivariate analysis.

#### 2.4.2. Assessment of Frailty Syndrome

Frailty syndrome was assessed according to Fried’s criteria. Five criteria were assessed, with a cut-off point of three or more of these for the diagnosis of frailty [43]. In addition, following Fried’s recommendations [43], those women who did not meet any criteria at the time of the assessments were considered robust, and those who met one or two criteria were considered pre-frail [43]. The five criteria with their respective cut-off points proposed by Fried were the following: (i) unintentional weight loss of 4.5 kg or more in the last year; (ii) self-reported exhaustion, assessed by the question “how often in the last week did you feel that everything you did caused you fatigue?” (this was considered positive when the participants’ response was “often” or “most of the time” and negative when it was “never” or “sometimes”); (iii) low physical activity, assessed with the International Physical Activity Questionnaire (IPAQ), validated in Spanish (this criterion was considered positive when the Kcal consumed per week of physical activity was less than 270); (iv) motor slowness, calculated using the 4.6 m walking test, with the cut-off point set at 6 s for participants with a height ≥1.59 m and at 7 s for participants with a height <1.59 m; (v) low muscle strength. The overall muscular condition was calculated based on the handgrip strength measured with a model dynamometer manufactured by Jaymar (Preston., Jackson, MS, USA). The cut-off points were adjusted according to the participants’ BMI, with a cut-off point of 17 or less considered positive for the women with a BMI of 23 or less, a cut-off point of 17.3 or less for those with a BMI of between 23.1 and 26, a cut-off point of 18 or less for those with a BMI of between 26.1 and 29, and a cut-off point of 21 or less for those with a BMI over 29 [43]. Frailty syndrome was evaluated by identifying 5 criteria: unintentional weight loss, muscle weakness (decreased grip strength assessed with a dynamometer), low energy or exhaustion, slow walking speed, and a reduced physical activity level. Based on Fried’s frailty syndrome definition, patients are classified as robust when they do not fulfill any criteria; pre-frail: 1–2 criteria; frail ≥ 3 criteria [43].

#### 2.4.3. Depressive and Anxiety Symptoms

The Goldberg Anxiety and Depression Scale, validated in Spanish [44], was used to measure the levels of anxiety and depression in our participants. This is a hetero-administered questionnaire with two subscales, one for anxiety and one for depression. Each of the subscales is structured into 4 initial screening items to determine whether or not a mental disorder is likely to exist, and a second group of 5 items that are asked only if positive answers are obtained to the screening questions (2 or more on the anxiety subscale, 1 or more on the depression subscale). The cut-off points are greater than or equal to 4 for the anxiety scale and greater than or equal to 2 for the depression scale. In the geriatric population, its use as a single scale has been proposed, with a cut-off point of ≥6. A previous study in a non-oncology population reported that the Spanish version of this scale had reliability assessed by Cronbach’s alpha coefficients of 0.73 for the anxiety subscale and 0.78 for the depression subscale, both of which were considered acceptable [45].

#### 2.4.4. Evaluation of Subjective Sleep Quality

The subjective sleep quality of patients was assessed using a validated tool: the Athens insomnia scale [46]. This self-administered psychometric tool has been validated in a Spanish population [47]. The Athens scale is divided into eight questions, five of which assess nighttime sleep, and the last three assess the respondents’ daytime functionality. Each question is scored from 0 (total absence of problems in that item) to 3 (maximum level of dissatisfaction or impairment in that item by the respondents). The cut-off point for the diagnosis of insomnia is 6 points or more on the scale [48]. In the literature, it has been reported that the Cronbach’s alpha coefficient for the Athens insomnia scale in the Spanish population was 0.89 and 0.78 [49] for patients and the general population, respectively.

### 2.5. Statistical Analysis

For the descriptive analysis, quantitative variables were presented with the mean and standard deviation (SD), and categorical variables with the absolute value and the corresponding percentage.

Regarding the bivariate analysis, the normality of the quantitative variables and their homogeneity of variances between the different groups analyzed were confirmed using the Kolmogorov–Smirnov test and Q-Q graphs and the Levene test, respectively.

In the inferential analysis, tests were performed between related samples (same women before and after the program) and between independent groups (women with different characteristics within the sample). Regarding the analyses carried out between related samples, parametric tests of paired means were used in the case that the quantitative variables had a normal distribution and homogeneity of variances, and the non-parametric Wilcoxon test when the variables did not have a normal distribution or did not have homogeneity of variances. In addition, qualitative variables were analyzed among themselves in this pre-post-analysis using the McNemar test to analyze the differences in dichotomous categorical variables and the marginal homogeneity test for categorical variables in which one of them had three or more categories. Regarding the analyses between independent groups, the Student’s *t*-test or ANOVA was used when the quantitative variables in question had a normal distribution (depending on whether two or more groups had qualitative variables), and the non-parametric Mann–Whitney U test or Kruskal–Wallis test in different cases (depending on whether two or more groups had qualitative variables).

Correlations between quantitative variables were performed. In the case of variables with a normal distribution, the Pearson correlation test was used, and when one of the two variables did not have a normal distribution, the Kendall Tau correlation was used.

We used linear regression models to examine the association between the various social and health factors that could be associated with the scores on the Goldberg depression, Goldberg anxiety, Athens, and FS Criteria subscales in a multivariable linear regression model. To do so, we selected the variables that were significant in the bivariate analysis and those that clinically have a proven relationship with these dependent variables with solid evidence. In addition, a multivariate regression analysis was carried out using Pillai’s Trace to analyze the relationship of these predictor variables with these outcome variables as a whole [50,51]. Unstandardized and Standardized beta coefficients, 95% confidence intervals (95% CI), and statistical significance (*p*) were estimated for each predictor variable. All statistical tests were considered statistically significant at the *p* < 0.05 level.

Data analysis was carried out using the SPSS version 29.0 statistical package (SPSS Inc., Chicago, IL, USA) licensed to the University of Valencia.

## 3. Results

### 3.1. Sociodemographic and Clinical Data

During the recruitment phase of the proposed multimodal program (September 2023 and April 2024), a total of 52 patients agreed to participate and performed both pre- and post-evaluations in four successive groups. The mean age of the 52 participants was 66.96 ± 5.05. The participants had an average time since their oncological BC diagnosis of 40.12 ± 31.55 months. Regarding the time they had been on hormonal treatment with AI, the average was 29.02 ± 23.89 months. Thirty-one of the women were married (59.6%), and 23.1% lived alone (Table 1). Regarding medication intake, 25 of the women (48.1%) took five or more medications per day; that is, they met the criteria for polypharmacy. Regarding the use of medications specifically related to mental health, nine women (17.3%) took anxiolytics, fifteen women (28.8%) took antidepressants, and seven women (13.5%) took sleeping pills. Regarding the type of AI they took, the most frequently used was anastrozole with 41 women (67.3%), and the least frequent was exemestane with 4 participants (5.8%). A high level of adherence to the program was obtained by women, both to exercise training (89.18% ± 10.03) and group talks and debates on health education (94.3% ± 11.3).

### 3.2. Changes in Frailty After the Program

There were significant differences in the number of FS criteria met between the beginning and the end of the program (*p* < 0.001). In particular, there was a mean reduction of 1.2 FS criteria (1.8 ± 1.01 pre and 0.4 ± 0.64 post). Analyzing each criterion separately and considering the cut-off points and conditions that make up the criteria (see Methods Section 2.4.2.), significant differences were observed for Weakness, Low physical activity, Slow gait speed, and Low muscle strength (Table 2). In the question about self-perceived weakness, 12 of the women answered “never” before the program, and 35 of the women did so afterward (*p* < 0.001). The mean obtained before and after the program in the tests evaluating Low physical activity (794.4 ± 837.39 pre and 2564.5 ± 1233.35 post; *p* < 0.001), Slow gait speed (4.93 ± 1.29 pre and 4.32 ± 0.86 post; *p* = 0.004) and Low muscle strength criteria (18.3 ± 4.58 pre and 21.7 ± 5.22 post; *p* < 0.001) also showed significant differences. No significant differences were found for Involuntary weight loss (*p* = 1) (Table 2).

**Figure 1:**
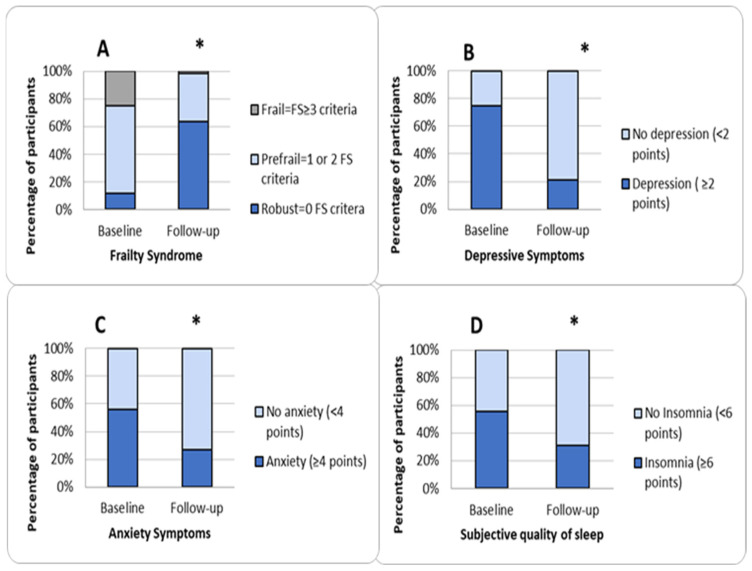
(**A**) Evaluation of frailty syndrome based on the presence of the number of criteria after the program compared at the baseline. FS: frailty syndrome. Classification as robust, pre-fragile or fragile was based on Fried’s classification of the physical phenotype of frailty syndrome [34] (see also Methods Section 2.4.2. Assessment of frailty syndrome). (**B**) Assessment of the diagnosis of depression according to the Goldberg depression subscale criteria before and after the program [44]. (**C**) Assessment of the diagnosis of anxiety according to the Goldberg anxiety subscale criteria before and after the program [44]. (**D**) Assessment of the diagnosis of insomnia according to the Athens subjective sleep quality scale criteria [46] (see also Methods Section 2.4.3. and Section 2.4.4.) (* significant difference obtained with the McNemar test; *p* < 0.001).

There was a significant difference in the number of robust, pre-frail and frail women after the program compared to the presence at the baseline. Six women did not fulfill any criteria for (robust) FS before the intervention (11.5%), and 33 women (63.5%) did so afterward. A total of 33 (63.5%) women met one or two FS criteria (pre-frail) before the intervention, 18 (34.6%) did so afterward, and 13 (25%) women met three or more FS criteria (frail) before the intervention, and 1 (1.9%) did so afterward (*p* < 0.001). (Figure 1A).

### 3.3. Changes in Mental Health After the Program

The total Goldberg scale score was significantly lower after the intervention compared to baseline (7.50 (7.00) before the program, 1.00 (5.00) after the program; *p* < 0.001) (Table 3).

Both the depression (3.00 (3.75) before the program, 0.00 (1.00) after the program; *p* < 0.001) and anxiety (5.00 (6.00) before the program 1.00 (5.00) after the program; *p* < 0.001) subscales showed significant changes after the intervention (Table 3).

With a cut-off point of two points or more on the depression subscale, 75.0% of the participants (n = 39) presented symptoms of depression before the program, and 21.2% (n = 11) did so after it (*p* < 0.001) (Figure 1B) (Table 4). With a cut-off point of four points or more on the anxiety subscale, 55.8% of the participants (n = 29) presented symptoms of anxiety before the program, and 26.9% (n = 14) did so after it (*p* < 0.001) (Figure 1C) (Table 4). For the total score on the scale, considering a value of six or more points, which is equivalent to the third quartile, 59.6% of the participants (n = 31) showed symptoms of anxiety and depression before the program, and 21.2% (n = 11) did so after the program (*p* < 0.001).

Significant differences were observed in the participants’ level of insomnia for both the average scores (Table 3) on the Athens scale and using the cut-off points proposed by the scale (6 or more points on the scale is considered insomnia). A total of 55.8% of the participants (n = 29) presented symptoms of insomnia before the program, and 30.8% (n = 16) did so after the program (Figure 1D) (Table 4) (*p* < 0.001).

### 3.4. Symptoms of Anxiety and Depression and Sleep Quality and Their Relationship with Sociodemographic and Clinical Characteristics

The scores on the Athens scale before the intervention were directly correlated with the score obtained before the program for both the overall Goldberg score (Kendall Tau = 0.414, *p* < 0.001) and the two Goldberg subscales of anxiety (Kendall Tau = 0.481, *p* < 0.001) and depression (Kendall Tau = 0.206, *p* = 0.046) obtained before the program (Table 5). These variables were correlated after the intervention as well (Table 6). Similarly, statistically significant differences were found for the Athens scale score between women who exceeded the cut-off point for the Goldberg anxiety subscale and those who did not (11.03, SD: 5.946 vs. 3.26, SD: 3.264 *p* < 0.001, Mann–Whitney Test). There were no statistically significant differences in the Athens scale score between women who exceeded the cut-off point for the Goldberg depression subscale and those who did not (8.46, SD: 6.456 vs. 5.0, SD: 5.0 *p* = 0.082, Mann–Whitney Test). Statistically significant differences were found for the total score on the Goldberg scale between the women who exceeded the cut-off point for insomnia on the Athens scale and those who did not (9.28, SD:3.844 vs. 4.70, SD: 4.050 *p* < 0.01, Mann–Whitney Test). The participants’ age was inversely correlated with the Goldberg anxiety subscale score (Kendall Tau = −0.217, *p* = 0.37) but not with the depression subscale (Kendall Tau = −0.61, *p* = 0.553), nor with the total Goldberg score (Kendall Tau = 0.167, *p* = 0.97). No significant correlations were found between the Goldberg scores and the time since diagnosis (for the anxiety subscale Kendall Tau = 0.189, *p* = 0.063), (for the depression subscale Kendall Tau = 0.067, *p* = 0.507), (for the total score Kendall Tau = 0.159, *p* = 0.106), or with the length of time the women had been on AI treatment (for the anxiety subscale Kendall Tau = 0.177, *p* = 0.082) (for the depression subscale Kendall Tau = 0.049, *p* = 0.626) (for the total score Kendall Tau = 0.155, *p* = 0.117), or between the BMI before the intervention or the number of drugs that the women were taking and the total Goldberg scale score. There were no differences in the total Goldberg score according to the patients’ dichotomized marital status (the rest 6.62, SD: 4.873 vs. married women 7.68, SD: 4.300 *p* = 0.280, Student’s *t*-test). The total Goldberg score was not associated with the type of AI (*p* = 0.370, Anova test), with the dichotomized stage of cancer at diagnosis (stage 1 6.93, SD: 4.088 vs. the rest 7.65, SD: 5.087 *p* = 0.167, Student’s *t*-test), with having received previous chemotherapy or not (7.00, SD: 4.370 vs. 7.42, SD: 4.689 *p* = 0.772, Student’s *t*-test), or with the dichotomized type of surgery (conservative surgery 7.45, SD: 4.691 vs. mastectomy 6.40, SD: 3.836 *p* = 0.433, Student’s *t*-test).

### 3.5. Multivariate Analyses

A linear regression analysis was conducted to examine the associations between the depression subscale score after the intervention and various predictor variables, including age, dichotomized marital status, percentage of attendance at training and health education sessions, whether or not the individual had received chemotherapy, time since diagnosis, time on AI treatment, type of dichotomized surgery received, the number of daily medications taken, and scores obtained before the program on the Goldberg anxiety subscale, the Goldberg depression subscale, the Athens insomnia scale, and the number of FS criteria (Table 7). The results indicated significant negative associations between the depression subscale score after the intervention and the percentage of attendance at training sessions (*p* = 0.041, Unstandardized beta coefficient = −0.018, 95% CI −0.104–−0.002); therefore, participants with a higher rate of adherence to the training had lower rates of depression after the program as well as a dichotomized marital status (*p* = 0.047, Unstandardized beta coefficient = −0.929, 95% CI −1.844–−0.14), with a negative association between married women compared to other women and the depression subscale score after the intervention. Significant positive associations were also observed with the score obtained before the program on the Goldberg depression subscale (*p* = 0.004, Unstandardized beta coefficient = 0.302, 95% CI 0.102–0.503). However, no significant associations were found with the other items, as shown in Table 7.

Three more multivariable linear regressions were carried out to determine the associations between the Goldberg anxiety subscale, the Athens scale, and the number of frailty criteria after the intervention and the same predictor variables as in the Goldberg depression subscale, but no significant associations were found with any of them except between the Athens scale score after the intervention and the score on the same scale before the intervention (*p* < 0.001, Unstandardized beta coefficient = 0.516, 95% CI 0.272–0.759).

A multivariate regression analysis was carried out to analyze the relationship between the predictor variables selected in the multivariable linear regressions and the different dependent variables (number of frailty criteria, the score of depressive, anxiety, and insomnia symptoms after the program as outcomes) of each of the linear regressions as a whole. There was a linear relationship between the Athens insomnia scale score before the program and all dependent variables (Goldberg depression subscale score after the program, the Goldberg anxiety subscale score after the program, the Athens scale score after the program, and the number of frailty criteria after the program) (Pillai’s Trace = 0.045, F = 6.408, *p* < 0.001). The rest of the independent variables did not show a statistically significant relationship with the set of dependent variables.

## 4. Discussion

This longitudinal study aimed to determine whether frailty levels, anxiety and depressive symptoms, and sleep quality were reduced in patients with localized breast cancer treated with aromatase inhibitors after 3 months of combined and supervised physical exercise and six group health education sessions.

Adherence to this type of intervention is key, especially in patients diagnosed with BC, who perform less physical exercise, are less motivated, and/or have less knowledge about exercise (and even worse in those patients with some criteria of frailty and/or with depressive or anxious symptoms) [24,52]. The loss of functionality is greater among older women who meet one or more frailty criteria than among younger or robust older patients [10,24,53]. It is to be expected that the decline in the levels of physical exercise in this group of women will be even more marked. The program was group-based and sought to improve perceived social support by improving the women’s capabilities and self-efficacy [54,55]. In addition, it was mostly carried out outdoors to increase the impact on mental health [53].

Due to the impact that a BC diagnosis (and its initial treatments) postmenopause and especially hormonal treatment with AIs have on the frailty levels of older patients, it is essential to slow the pace of this deterioration or even improve this aspect through physical exercise interventions based on robust evidence.

Our multimodal intervention showed significant effects in this regard. A significant reduction in the number of frail and pre-frail women was achieved after the program, as well as an average reduction in the number of criteria before and after the program and a reduction in the count of each of the criteria on the FS (except for involuntary weight loss). These results are supported by studies that evaluated frailty levels before and after a physical exercise intervention in oncological patients with some methodological differences compared to our study, such as not recording whether they took AIs [26] and not limiting the study to postmenopausal women [56].

Despite not evaluating FS as a whole, other publications have analyzed the impact of resistance training programs on fatigue in this specific group of patients and support these findings [57,58,59]. A 12-month telephone-based exercise intervention did not reduce functional decline at one year but provided preliminary evidence that may prevent physical performance decline at two years in older adults with breast cancer [29]. At baseline, only five women (9.6%) met the FS criterion of unimproved unintentional weight loss after the intervention. This prevalence is consistent with the non-significant impact of AIs on this specific criterion in other studies. Our findings for gait speed and the International Physical Activity Questionnaire score are consistent with those obtained with exercise interventions in the BC subgroup [29]. In this case [29], the deterioration of oncogeriatric patients with BC slowed down after a long-term intervention but not in other types of cancer.

Meanwhile, in regard to mental health, in line with the benefits obtained after the program, the beneficial impact of physical exercise (especially combined and supervised) and health education programs on depressive and anxious symptoms and on subjective sleep quality among women with BC and postmenopausal women receiving AI treatment, in particular, has been extensively described [42,60,61]. These results differ from those of the exercise intervention in women diagnosed with BC receiving AI treatment who did not show any improvement in their depressive symptoms [58]. This intervention assessed depressive symptoms (among other health issues) with a different instrument (the Finnish-modified version of Beck’s 13-item depression scale) 5 years after the start of a 12-month intervention. This intervention was also carried out with both postmenopausal and premenopausal women.

The results of the linear regression showed marital status and the percentage of attendance at health training sessions (in addition to the score on the Goldberg depression subscale before the program) as predictive variables for a lower score on the Goldberg depression subscale after the program. Marital status was equivalent to cohabitation among the participants since there were no women living with partners without being married. The married women showed lower scores on the Goldberg depressive symptomatology subscale after the intervention than their single, divorced, or widowed counterparts. This association is consistent with previous studies addressing the relationship between marital status, self-care, and self-reported health [62]. Furthermore, the protective role of being married that promotes better outcomes in interventions in cancer patients has been previously reported [28,63], and it is probably related to the greater social and emotional support this entails, facilitating the maintenance of healthy lifestyles among older patients. In regard to attendance at training sessions, other than increased compliance with the planned exercise schedules, people who attended training more frequently also obtained greater benefits from the social impact of the program. Low levels of social support, as one of the factors associated with depressive symptoms in breast cancer survivors, must be taken into account in interventions that aim to improve these patients’ mental health [64]. When analyzing the outcomes, e.g., the scores of depressive, anxiety, and insomnia symptoms and FS Criteria after the program in a multivariable linear regression model, we found that the variable that significantly predicted those outcomes was the quality of sleep before the program. This results is supported by literature showing that sleep quality and frailty syndrome are closely related to each other [65], so it is conceivable that the program increased both sleep quality and improved frailty. Older adults with sleep disorders may experience adverse outcomes such as decreased grip strength, fatigue, and slow walking speed, which are typical symptoms of frailty [66,67]. In the case of the predictive effect of sleep quality before the program on the improvement of depressive and insomnia symptoms after the program, there is a huge body of evidence that these mental health symptoms are closely related. For example, poor sleep quality could predict depressive symptoms [68], and both physical inactivity (a criterion of frailty syndrome) and poor sleep quality interact to predict depressive symptoms [69]. Confirming this relationship, improvements in sleep quality can predict antidepressant drug response and are linearly correlated with improvements in overall depressive symptom severity [70]. Other types of intervention support the predictive value of sleep quality at the baseline of depressive symptoms after interventions to improve mental health quality, such as group cognitive behavioral therapy [71].

Neither the time of hormonal treatment nor the type of AIs were significant in the number of frailty criteria after the program or in the Goldberg anxiety, Goldberg depression, or Athens scores. Although there is evidence to suggest that AIs increase frailty levels in postmenopausal patients diagnosed with BC [11], these results do not take into account the level and type of physical exercise these women engage in, which, as our findings suggest, could have a moderate impact. In addition, this study [11], like ours, did not use a control group of patients with the same diagnosis and the same characteristics who were not receiving treatment with AI in order to be able to analyze the social and emotional impact of the cancer diagnosis, and of the other treatments, separately from the impact of AIs. None of the sociodemographic and clinical variables were associated with the levels of frailty, anxiety, or insomnia after the intervention, suggesting that other unknown factors could be related, such as the role of comorbidities or the specific drugs used to treat them. The small sample size of this study meant it was impossible to study the influence of specific comorbidities on the study results or the impact of previous chemotherapy [72], which was present in only just over half of the study sample (59.6%).

In this group of cancer survivors, the frailty index is associated with worse mental health, QoL, and survival outcomes (both due to the cancer diagnosis and from all causes) [73,74,75,76]. For all the above reasons, and due to the overlap between these entities, physical exercise should be used in this group of patients as a continuous tool from the time they enter the service at the time of diagnosis and whenever possible, even if it means adapting the loads and type of exercises during and after the therapeutic phases, with the aim of improving the physical and psychological health of the patients and increasing their life expectancy [61].

Increasing the levels of evidence for this type of physical exercise intervention (with their different types of exercise, multimodal or non-multimodal proposals, supervised or not, etc.) for older adults with hormone-dependent breast cancer can help improve specific cancer guidelines and build an evidence base that enables these programs to be implemented in a widespread and non-opportunistic manner.

## 5. Limitations

The present study has some important limitations that should be taken into account. The first limitation is the small sample size and the single-center nature of the study. However, the size was considered sufficient to extrapolate the results to the characteristics of patients with localized breast cancer receiving adjuvant treatment. However, this sample size may have led to significant improvement results in the implementation of this intervention on a larger scale to improve frailty and psychological well-being among these patients. The intervention was conducted with women with very long exposure to AI treatment, and no comparisons were made with a control group that did not undergo the intervention. Finally, although it facilitated adherence and program cohesion, the participant’s knowledge of the program objectives and the fact that some of the investigators conducted the interviews can also be considered an important bias. Additional interventions with larger samples, control groups, and long-term follow-up would be useful to validate these results and assess levels of improvement over time.

However, although randomized controlled trials allow us to reach more specific conclusions about the intervention, there is now some consensus about the utility of alternative clinical research methods that can complement the findings of RCT designs and improve our ability to evaluate the efficacy of treatments for individual patients with a more personalized medicine approach. These investigations usually include samples with a smaller N but are characterized by serial observations of individual people or small groups before, during, and after an intervention period. This patient-level approach can facilitate evidence-based medicine in two general ways. First, it allows us to provide practical information for making decisions individualized according to the characteristics of each patient. Second, it allows us to reach conclusions without having the very strict inclusion criteria of RCTs and allows us to describe beneficial health effects in more ecological real-life treatment environments, such as in the case of the patients in this study who lived in the community [77,78]. The focus on individual patients is an important element of evidence-based rehabilitation, and having selected women with frailty and/or psychological alterations in our trial can support this new approach.

As an additional limitation of the study, we have not validated the scales used in our study, e.g., exploratory factor analysis or confirmatory factor analysis; however, in the case of the Athens insomnia scale, it has already demonstrated good construct validity for insomnia and Fried frailty criteria in oncology patients [79,80]. The internal consistency and temporal stability have been validated in oncology patients [79,81]. Moreover, the two-component factor structure is clear and stable between different cancer diagnoses, and either exploratory, confirmatory, or multigroup analyses confirmed these findings [82]. In the case of the Goldberg anxiety and depression scale, there is a need to validate its validity, and this aspect warrants future research; however, all the instruments have been previously validated in community-dwelling older individuals with similar age to those of our sample [83,84,85], suggesting they are appropriated for this older population. The Fried frailty scale, Athens insomnia scale, and other scales of anxiety and depression symptoms have been validated in oncology patients with similar results as those obtained in the general population [84,86,87,88].

## 6. Implications for Practice

Given the high prevalence of frailty in postmenopausal cancer patients during hormone treatment [11], it seems clear that the healthcare team needs to systematically assess the levels of frailty in these patients before, during, and after taking this type of drug.

Similarly, the bidirectional relationship between frailty levels and mental health, on the one hand [10,23,24,25,26,70], and hormone therapy and anxiety, depression, and sleep quality symptoms, on the other [11,14,15,20,21], requires an assessment of these health areas as an initial screening and evaluation during the follow-up of these patients.

Physical exercise, accompanied by good health education, can help to reduce the levels of frailty in women on hormone treatment and also improve their levels of anxiety, depression, and insomnia [60,61].

Nurses play a fundamental role in health education in older cancer patients, helping them acquire knowledge about hormone therapy in general and AI inhibitors in particular, which are key drugs in the prevention of breast cancer relapses, in order to increase adherence to these drugs and prolong their prescription for longer periods while maintaining a good quality of life.

The implementation of multimodal programs carried out by interdisciplinary teams could have a positive impact on their physical and mental health, reduce the unwanted effects of AI inhibitors, and motivate these patients to improve their self-care and increase their knowledge.

## 7. Conclusions

Frailty syndrome and psychological alterations are common among postmenopausal women diagnosed with BC receiving hormonal treatment with AI. These physical and emotional symptoms can be improved with a multimodal program combining supervised physical exercise and health education workshops. This program should be incorporated into the rehabilitation protocols of BC patients after acute treatment in order to prevent adverse outcomes associated with frailty and psychological complaints.

## Figures and Tables

**Table 1:** Demographic and clinical characteristics.

Demographic Variables	Total Number of Women Starting the Program (n = 52)
Age, years	
Mean (SD)	66.96 ± 5.05
Range	60–78
Employment status	
Retired	33 (63.5%)
Active	4 (7.7%)
Unemployed	15 (28.8%)
Marital status	
Married	31 (59.6%)
Widowed	7 (13.5%)
Divorced	7 (13.5%)
Single	7 (13.5%)
Medical variables	
BC Stage	
IA	30 (57.7%)
IIA	11 (21.2%)
IIB	11 (21.2%)
Previous chemotherapy	
Yes	21 (37.5%)
No	35 (62.5%)
Type of surgery	
Conservative	44 (78.6%)
Mastectomy	12 (21.4%)

Values represent mean (SD) or frequencies (n, %).

**Table 2:** Frailty syndrome criteria count at baseline and after the program.

Outcome Variable	Baseline: Prevalence (%)		12 Weeks: Prevalence (%)		*p*-Value
	No	Yes	No	Yes	
Involuntary weight loss	47 (90.4%)	5 (9.6%)	48 (92.3%)	4 (7.7%)	1
Weakness	31 (59.6%)	21 (40.4%)	47 (90.4%)	5 (9.6%)	<0.001 *
Low physical activity	32 (61.5%)	20 (38.5%)	51 (98.1%)	1 (1.9%)	<0.001 *
Slow gait speed	42 (80.8%)	10 (19.2%)	51 (98.1%)	1 (1.9%)	0.004
Low muscle strength	18 (34.6%)	34 (65.4%)	38 (73.1%)	14 (26.9%)	<0.001 *

* obtained with the McNemar test.

**Table 3:** Average score on the Goldberg and Athens mental health scales and estimated differences in *p* values.

	Baseline: Median (IQR)	12 Weeks: Median (IQR)	*p*-Value
Total Goldberg scale	7.50 (7.00)	1.00 (5.00)	<0.001 *
Goldberg subscales			
Anxiety subscale	5.00 (6.00)	1.00 (5.00)	<0.001 *
Depression subscale	3.00 (3.75)	0.00 (1.00)	<0.001 *
Athens scale	6.00 (9.75)	4.00 (6.75)	<0.001 *

* Obtained with the Wilcoxon test. (IQR: Interquartile Range).

**Table 4:** Count of depression, anxiety, and insomnia criteria at baseline and after the program.

Outcome Variable	Baseline: Prevalence (%)		12 Weeks: Prevalence (%)		*p*-Value
	No	Yes	No	Yes	
Goldberg anxiety subscale	23 (44.2%)	29 (55.8%)	38 (73.1%)	14 (26.9%)	<0.001 *
Goldberg depression subscale	13 (25.0%)	39 (75.0%)	41 78.8%)	11 (21.2%)	<0.001 *
Athens scale	23 (44.2%)	29 (55.8%)	36 (69.2%)	16 (30.8%)	<0.001 *

* Obtained with the McNemar test.

**Table 5:** Correlation matrix between the dependent variables of the study before the program.

Variables	1	2	3	4
Number of Frailty Syndrome criteria (1)	-			
Goldberg anxiety subscale (2)	−0.28	-		
Goldberg depression subscale (3)	0.174	0.332 **	-	
Athens insomnia scale (4)	0.037	0.481 **	0.206 *	-

Obtained with the Kendall Tau test. * = *p* < 0.05 (bilateral) ** = *p* < 0.01.

**Table 6:** Correlation matrix between the dependent variables of the study after the program.

Variables	1	2	3	4
Number of Frailty Syndrome criteria (1)	-			
Goldberg anxiety subscale (2)	0.283 *	-		
Goldberg depression subscale (3)	0.301 *	0.438 **	-	
Athens insomnia scale (4)	0.243 *	0.501 **	0.327 **	-

Obtained with the Kendall Tau test. * = *p* < 0.05 (bilateral) ** = *p* < 0.01.

**Table 7:** Multivariable linear regression model: Association of the Goldberg depression subscale with predictive clinical variables.

Variables	Unstandardized Coefficient		Standardized Beta Coefficient	t	*p*-Value	95% CI	
	B	Std Error				LL	UL
Age	0.020	0.044	0.056	0.464	0.645	−0.68	0.109
Marital status marriage (ref: No marriage)	−0.929	0.452	−0.249	−2.055	0.047 *	−1.844	−0.14
% of training sessions attended	−0.018	0.011	−0.279	−2.111	0.041 *	−0.104	−0.002
% of health education sessions attended	0.004	0.021	0.028	0.188	0.852	−0.038	−0.046
Number of different medications taken	0.113	0.077	0.182	1.462	0.152	−0.043	0.268
Time since oncological diagnosis	−0.007	0.011	−0.121	−0.623	0.537	−0.030	0.016
Time with AI	0.001	0.016	0.019	0.095	0.925	−0.030	0.033
Chemotherapy yes (ref: No)	0.644	0.463	0.173	1.391	0.172	−0.293	1.581
Type of surgery mastectomy (ref: conservative)	−0.515	0.642	−0.111	−0.802	0.427	−1.814	−0.785
Goldberg anxiety subscale	0.120	0.119	0.187	1.009	0.319	−0.121	0.360
Goldberg depression subscale	0.302	0.099	0.395	3.056	0.004 *	0.102	0.503
Athens insomnia scale	0.003	0.056	0.011	0.060	0.952	−0.109	0.116
Number of FS criteria	0.437	0.246	0.238	1.776	0.084	−0.061	0.936

(AI: Aromatase Inhibitors; FS: Frailty syndrome; B: Unstandardized beta coefficient; Std Error: Standard Error; LL: lower limit; UL upper limit). R = 0.759, R squared = 0.575, Adjusted R squared = 0.430. Anova F-Test: 3.961. Anova *p* value < 0.001. * indicates statistical significance at *p*-value < 0.05. The Goldberg depression subscale score as a variable to predict: the higher the score, the greater the depressive symptoms shown.

## Data Availability

The data presented in this study are available from the corresponding author on request.
